# Development of an Equivalent Analysis Model of PVB Laminated Glass for TRAM Crash Safety Analysis

**DOI:** 10.3390/polym17010025

**Published:** 2024-12-26

**Authors:** Yuhyeong Jeong, Youngjin Jeon, Wonjoo Lee, Jonghun Yoon

**Affiliations:** 1 Department of Mechanical Engineering, Hanyang University ERICA, 55, Hanyangdaehak-ro, Sangnok-gu, Ansan-si 15588, Gyeonggi-do, Republic of Korea; jtpye9402@gmail.com (Y.J.); jj981226@hanyang.ac.kr (Y.J.); wj6478@gmail.com (W.L.); 2 Department of Mechanical Engineering, BK21 FOUR ERICA-ACE Center, Hanyang University ERICA, Ansan-si 15588, Gyeonggi-do, Republic of Korea

**Keywords:** head injury criterion, equivalent model, PVB laminated glass, numerical analysis, dynamic head impact test

## Abstract

This study focuses on an equivalent model of Polyvinyl Butyral (PVB) laminated glass to simulate the Head Injury Criterion (HIC) when a pedestrian collides with a TRAM. To simulate the collision behavior that occurs when a pedestrian’s head collides with PVB laminated glass, a comparison was made between the results of the widely used PLC model for PVB laminated glass modeling and an actual dynamic head impact test. The material properties of the tempered glass and PVB film used in the PLC and equivalent models were obtained via four-point bending tests and tensile tests, respectively. The proposed equivalent model is developed by assigning the thickness, material properties, and positional information of each layer in the multilayer PLC model to the integration points of the shell element. The results of the equivalent analysis model were found to accurately simulate the collision behavior when compared with the results of both the dynamic head impact test and the PLC model. Moreover, the analysis cost improved to approximately 15% of that of the traditional PLC model.

## 1. Introduction

The TRAM is a type of rail vehicle, consisting of either individual railcars or self-propelled trains coupled into multiple units that run on tramway tracks on public roads. There is an increasing trend in the introduction of trams, driven by the growing demand for eco-friendly energy. Unlike conventional rail vehicles that run on designated railroads, trams operate on public roads, raising significant safety concerns not only for the vehicle itself but also for the objects it may collide with, particularly pedestrians [1,2,3,4,5,6]. Additionally, due to the vertically designed windshield shape [7,8], the collision behavior between a tram and a pedestrian shows a different pattern compared to that of a typical automobile and pedestrian collision. Therefore, the design of the windshield for trams requires a unique impact analysis model specifically for tram windshields, rather than relying on the collision behavior models of conventional rail vehicles or automobiles.

The front glasses of most automobiles, including trams, are primarily composed of Polyvinyl Butyral (PVB) laminated glass [9,10,11,12]. This material combines tempered glass with a PVB film to achieve the high strength of tempered glass while the PVB film prevents glass fragments from scattering during collisions. Due to these characteristics, PVB laminated glass not only ensures sufficient strength for vehicle safety but also prevents secondary damage caused by glass fragments during collisions. For this reason, it is widely used in vehicle windshields. Consequently, analysis methods that consider collisions, particularly those involving pedestrians, are well-established. For analyzing the effects of pedestrian-vehicle collisions, dynamic head impact tests are widely utilized. These tests simulate a pedestrian’s head using a dummy head, which is impacted against actual vehicles or automotive glass. By analyzing pre- and post-impact velocity and acceleration, the test evaluates the force and impulse exerted on the dummy head. This makes it an essential consideration in designing vehicles to minimize risks in pedestrian collisions.

Henn [13] analyzed the head injury criterion (HIC), which measures the likelihood of head injury from an impact, by comparing real crash tests using Mercedes–Benz cars. They calculated HIC parameters with and without airbags to compare injury risks, and the HIC became the standard by which to judge critical injury to a pedestrian’s head during a collision. Bois et al. [14] proposed a numerical analysis to simulate the crash behaviors of laminated safety glasses via computational analysis. They presented a laminated safety structure consisting of glass layers modelled with shell elements and PVB interlayers with membrane elements. In addition, they conducted spherical impact simulations and validated their model by comparing their results with those of a single-material model. Timmel et al. [15] modelled PVB laminated glass as a multilayer structure with solid and shell elements, conducted ball drop analyses, and compared the results with those of actual windshield drop tests. They also verified the consistency of the time–acceleration graphs, which are crucial for measuring the HIC parameters, between the experiments and analyses. Most studies model PVB laminated glass as multiple layers, considering glass layers as shell elements and PVB layers as solid elements, and apply the respective material properties to each layer. They predominantly verified the analysis models by comparing the fracture patterns and HIC parameter-influencing time–acceleration graphs resulting from the impacts of spherical rigid bodies simulating human heads against PVB laminated glass. However, from the perspective of TRAM design, glass often does not break upon collision with pedestrians, owing to the low operational speed of TRAMs. Therefore, front glass designs are required to minimize pedestrian injuries in noncracking situations. Although existing multilayer analysis models effectively simulate the acceleration values applied to a pedestrian’s head during a collision and the occurrences of cracks in glass, they require relatively long analysis times and high complexity in re-modeling every layer when modifications are needed. Consequently, such models are unsuitable for applications in the initial design step. Therefore, a new analysis model that is easier to modify, has lower analysis costs, and does not heavily consider glass breakage is needed.

Hence, this study proposes an equivalent analysis model that simplifies the multilayer structure of PVB laminated glass into a single-layer model. The proposed model maintains predictive accuracy comparable to experimental results and traditional multilayer models while dramatically reducing analysis costs to 15%, achieving approximately seven times the efficiency. This efficiency enables the rapid generation of pedestrian collision data specific to TRAMs, whose windshield is nearly vertical, aiding safety standard development. Additionally, it significantly reduces time and costs during frequent design iterations in the early design stages.

## 2. Experiments and Methodologies

### 2.1. Acquiring Material Properties of PVB Laminated Glass

**Figure 1:**
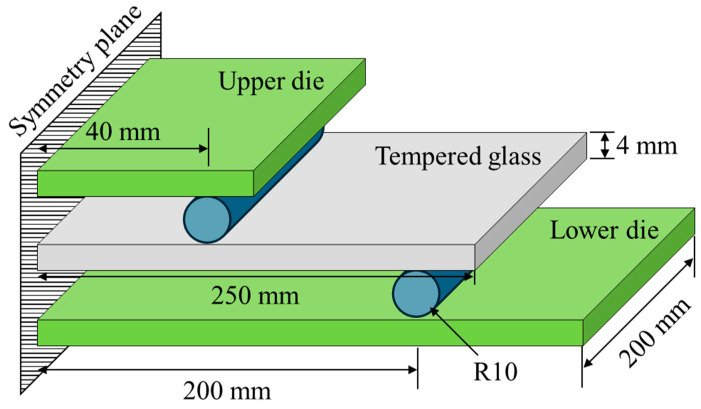
Schematic of the 4-point bending test, following the scaled ISO11228-3 standard.

**Figure 2:**
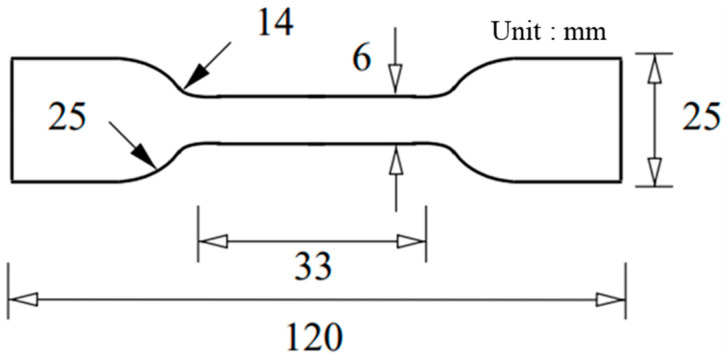
Schematic of a tensile test specimen for a PVB film, following the ISO573-3 standard.

**Figure 3:**
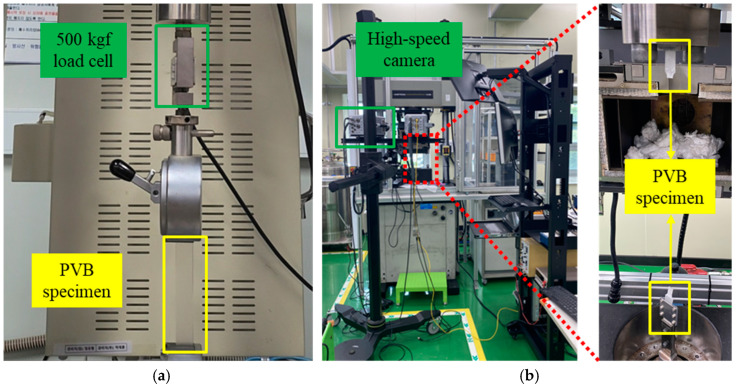
Experimental setup for a PVB film tensile test: (**a**) quasi-static strain rate; (**b**) high strain rate.

The material properties of the tempered glass and PVB film were experimentally obtained to construct an analytical model of the PVB-laminated glass. In addition, 4-point bending tests were conducted on tempered glass to investigate the effect of the strain rate. Experiments were conducted at three different strain rates (0.001, 0.01, and 0.1/s). Tempered glass cannot undergo additional post-processing because of its brittleness. Therefore, the experimental die set was produced by scaling the ISO 11228-3 standard [16] 4-point bending die set to fit the size of the prepared tempered glass, as shown in Figure 1. The PVB film material properties were obtained via quasi-static and high-speed tensile tests that were conducted at various strain rates (0.004, 1, 10, 100, 200, and 400/s) to consider the high strain rate during impact. Figure 2 shows a schematic of the tensile test specimen, following the ISO 573-3 standard. Quasi-static tensile tests were performed using a universal testing machine (UTM), as shown in Figure 3a, whereas high-speed tensile tests were conducted using a high-speed teTnsile testing machine available at the Korea Institute of Materials Science (KIMS), as depicted in Figure 3b. The strain was measured via the 2D digital image correlation (DIC) method. The material properties of the PVB film were obtained based on the strain rate, and fitting was performed using the Mooney–Rivlin fitting equation.

**Figure 4:**
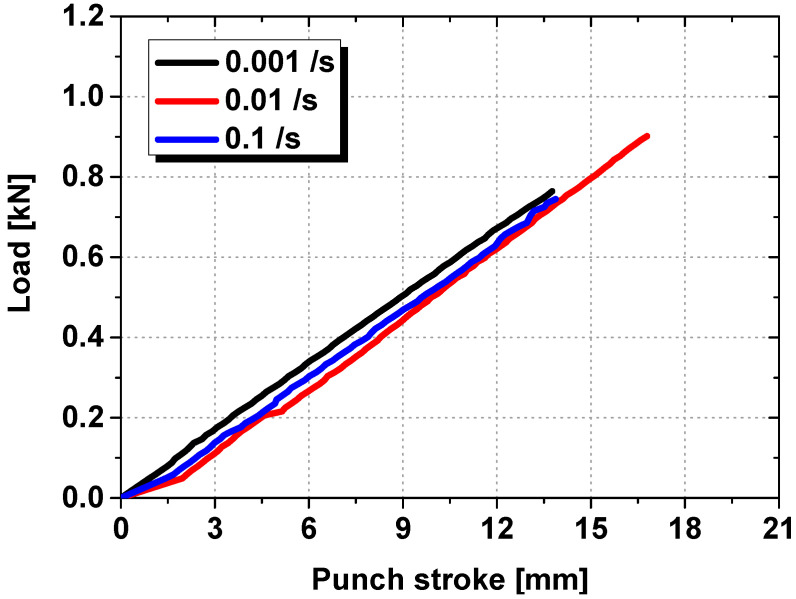
Punch stroke–load graph of tempered glass with respect to the strain rate.

Figure 4 shows the results of the 4-point bending test of the tempered glass. It appears that there is no change in the modulus with respect to the strain rate, which is attributed to the highly brittle nature of tempered glass and results in minimal strain rate dependency. Therefore, the material properties of the tempered glass applied in both the multilayer and equivalent models did not consider the strain rate effect. In contrast, a significant strain rate dependency was observed for the PVB film, which is a hyperelastic material that displays nonlinear behavior. The Mooney–Rivlin fitting equation, which is widely used to fit the stress–strain curves of hyperelastic materials, was used to fit the stress–strain data of the PVB film. The Mooney–Rivlin fitting equation is given by Equation (1):

(1) $$\sigma =2\lambda (1-\lambda^{-3})(A_{1}\lambda +A_{2}+A_{3}(\lambda^{2}+2\lambda^{-1}-3+\lambda (\lambda^{-2}+2\lambda -3))$$

λ=1+ε (stretch ratio)

$$A_{1},A_{2},A_{3}:\;\mathrm{Material}\;\mathrm{constants}$$

**Figure 5:**
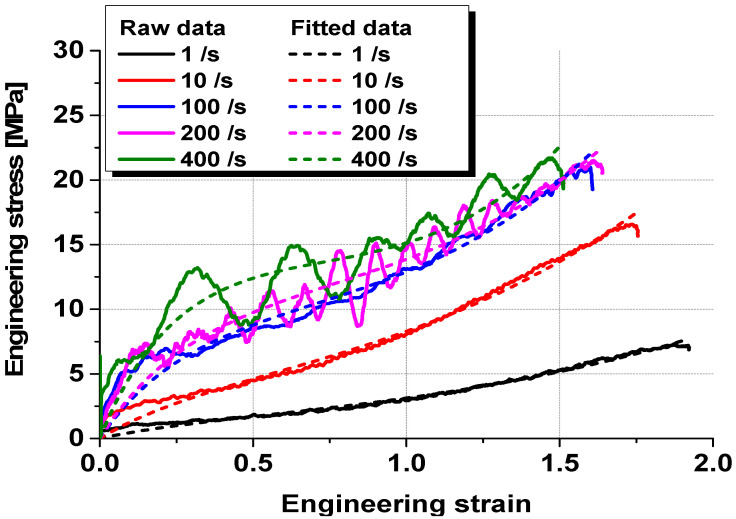
Stress–strain curves of PVB film with raw and fitted data, obtained via the Mooney–Rivlin equation.

The material constants for each strain rate were derived by fitting the stress–strain curve of the PVB film obtained from the tensile tests at different strain rates, as shown in Table 1. Figure 5 presents both the raw stress–strain data from the tensile test and data fitted using the Mooney–Rivlin equation. The fitted data were applied as material properties that accounted for the strain–rate dependency in the numerical analysis model.

### 2.2. Dynamic Head Impact Test

**Figure 6:**
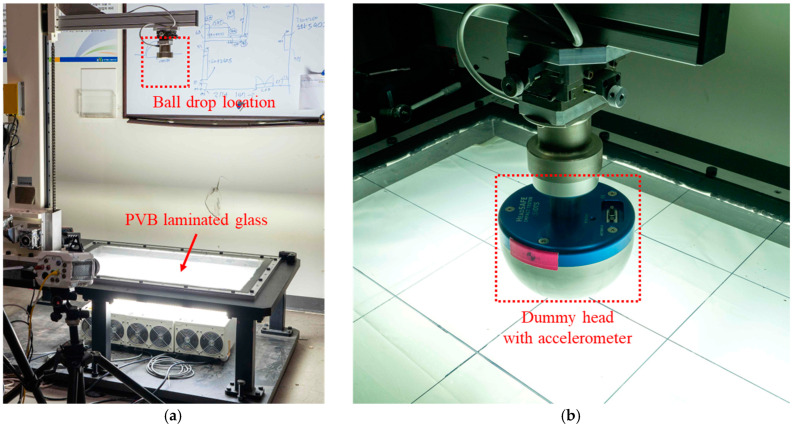
Experimental settings for the dynamic head impact test: (**a**) test jig for the dynamic impact test; (**b**) dropped ball, including the accelerometer.

**Figure 7:**
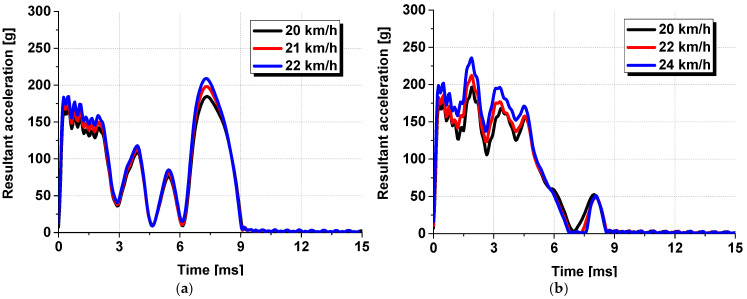
Time–acceleration results from the dynamic head impact test: (**a**) center impact results; (**b**) side impact results.

A dynamic head impact test was conducted using the free-fall method with a ball equipped with an internal accelerometer. PVB-laminated glass measuring 1000 mm in width, 700 mm in height, and 9.22 mm in thickness was manufactured for the experiment. The glass was attached to 30 mm wide and 4 mm thick rubber frame packing and fixed to a test jig using bolts. Figure 6 shows the PVB-laminated glass attached to the dynamic head impact test jig and ball for the free-fall. The experiment was conducted for six cases, using two different impact locations and three impact speeds. The impact locations were the center impact condition, for which the ball was dropped on the exact center of the PVB-laminated glass, and the side impact condition, for which the ball was shifted 325 mm from the center along the longer direction of the glass. The impact speeds for the center impact were set at 20, 21, and 22 km/h, and those for the side impact were 20, 22, and 24 km/h. These speeds were determined by converting the drop heights of the Dynamic Head Impact Test equipment into equivalent velocities. The experiments were conducted by adjusting the drop height to control the corresponding impact speed. After the free-fall of the ball, the accelerometer was removed from the ball, and the time-acceleration data were extracted at the moment of impact between the ball and PVB laminated glass. Figure 7 presents the time–acceleration graphs obtained from the center impact and side impact experiments. The detailed experimental conditions are also described in Ahn et al. [17].

## 3. Numerical Model for PVB-Laminated Glass

### 3.1. PVB-Laminated Glass Modeling with a Nonlocal Failure Criterion

**Figure 8:**
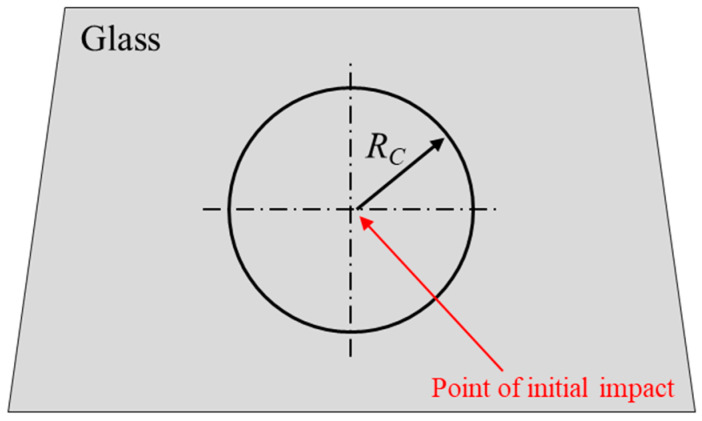
General parameters of the nonlocal approach.

The PLC criterion, which Pyttel et al. [18] called the nonlocal criterion, is an effective model for simulating the fracture behavior of glass caused by the impact between PVB-laminated glass and a pedestrian’s head. Owing to its highly brittle nature, tempered glass exhibits nonlinear fracture characteristics in which cracks propagate at a high speed once a certain point of external impact is reached. Consequently, accurate simulations using general fracture methods are impossible. Hence, an energy-based threshold criterion was added to the main criterion. If this criterion is not met, no fractures occur. Pyttel et al. determined the fracture occurrence threshold based on the energy accumulation of elements within a certain radius from the point where the initial impact occurs on the glass. Figure 8 illustrates the initial impact point on the glass and the radius of the circle used to calculate the energy accumulation around that point. The first impact between the ball and tempered glass follows the major stress condition represented in Equation (2):

(2) $$\sigma_{fail}=max\left(\left|\sigma_{1}\right|,\left|\sigma_{2}\right|\right)$$

The first impact area then becomes the center of the circle, using a predefined radius ($R_{C}$). The strain energy inside the finite region is calculated at each time step for each element.

The strain energy for each time step (*i*) and each element (*e*) can be represented as shown in Equation (3):

(3) $$\Delta E_{i}^{e}=\iint \sigma :d\dot{\varepsilon }dV^{e}\Delta t_{i}$$

The total strain energy in the impact area at time $t_{n}$ is represented by Equation (4):

(4) $$E^{e}\left(t_{n}\right)=\sum_{i=0}^{i=n}\Delta E_{i}^{e}$$

If the accumulated strain energy in the region exceeds the critical value, then the failure criterion in Equation (2) is activated for each element of the entire glass area. The critical value ($E_{C}$) was predefined based on the experiments:

(5) $$E>E_{c}$$

**Figure 9:**
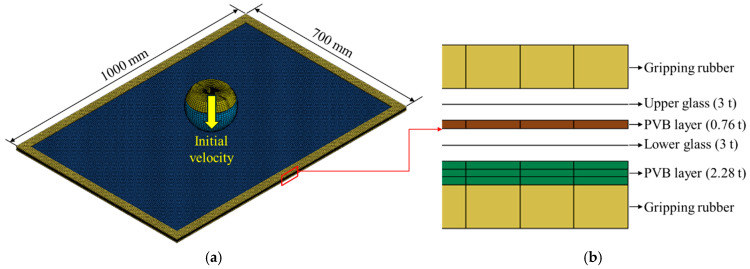
Numerical analysis model for the dynamic head impact test: (**a**) overall modeling; (**b**) structure of PVB-laminated glass in the direction of thickness.

**Figure 10:**
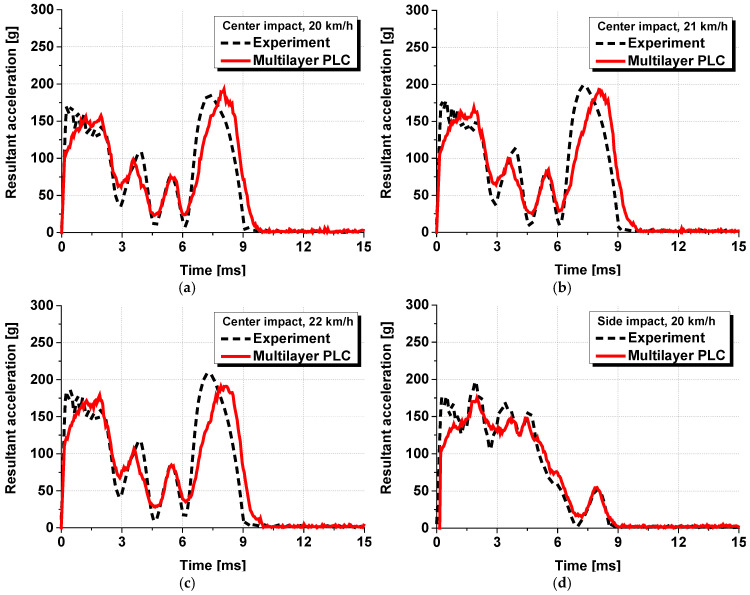

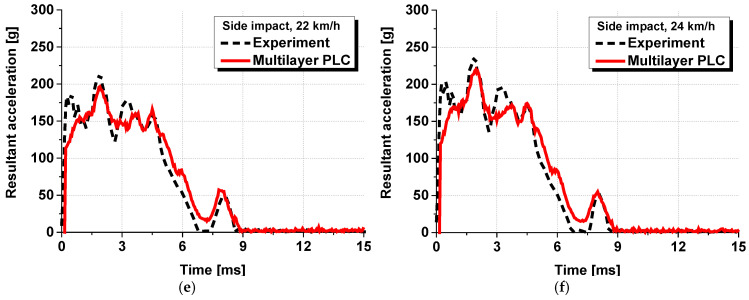
Analytical results of the dynamic head impact test with respect to the impact location and speed: (**a**) center impact at a speed of 20 km/h; (**b**) center impact at 21 km/h; (**c**) center impact at 22 km/h; (**d**) side impact at 20 km/h; (**e**) side impact at 22 km/h; (**f**) side impact at 24 km/h.

Using the above method, a ball-drop analysis was applied to the PLC fracture model using LS-DYNA 2021 R1 version, based on the widely used multilayer modeling method. The model was constructed under the same experimental conditions as those used in the dynamic head impact test. The tempered glass and PVB film layers of the PVB-laminated glass were modeled separately and combined by applying node–node tie conditions. For each gripping rubber, hard contact conditions were applied to the parts in contact with the glass, using a dynamic friction coefficient of 0.2 and a static friction coefficient of 0.3, whereas the nodes on the opposite side were fixed using a fixed boundary condition. Figure 9 shows a model of the dynamic head impact test. For the glass, a simple linear elastic model was used based on the experimental results in Section 2.1, and mechanical properties from the European standard DIN EN 572-1 [19] were applied. These values included a Young’s modulus of 70 GPa, Poisson’s ratio of 0.23, and density of 2.53 kg/m^3^. The material properties of the PVB film were determined by tabulating the stress–strain curves based on the strain rate. Figure 10 shows the analytical results of the dynamic head impact test and the experimental results. The analytical results indicate that the acceleration trend increases and decreases, closely following the experimental data.

### 3.2. Equivalent Model for PVB-Laminated Glass

**Figure 11:**
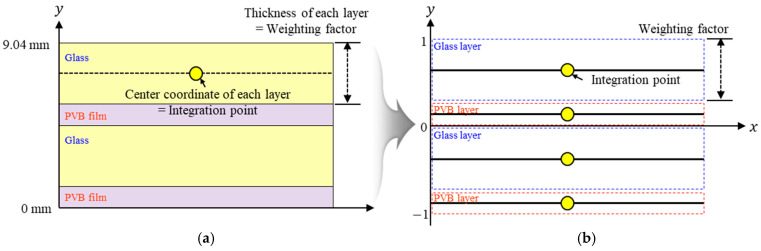
(**a**) Structure of PVB-laminated glass; (**b**) numerical structure of the equivalent model.

A single-layer structure was modelled for the equivalent model, including the material properties of the tempered glass and PVB film. In the proposed model, the integration points used in the shell elements were used to simulate each layer of the multilayer model. The location, thickness, and material properties of each integration point were controlled to simulate the multilayer structure; therefore, it played a role similar to that of the multilayer model. In LS-DYNA, the positions and thicknesses of each integration point should be converted to scaled values, with the total thickness of the structure being normalized to a range from −1 to 1. Thus, the total thickness of the PVB-laminated glass, that is, 9.22 mm, was normalized to 1, and 0 mm was normalized to −1. Then, the center points of each tempered glass and PVB film layer were calculated and applied. However, due to the single-layer structure of the proposed equivalent model, it is difficult to directly apply the failure criteria specific to the glass part in the PLC model. As a result, the equivalent model cannot simulate glass fracture behavior. Nonetheless, dynamic head impact tests considering TRAM operating speeds demonstrated no fracture of the PVB laminated glass, validating the equivalent model’s applicability. Figure 11 illustrates the structure of the PVB-laminated glass and numerical structure of the equivalent model simulated using integration points.

**Figure 12:**
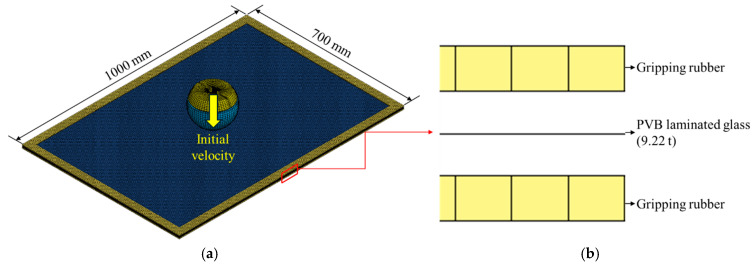
Numerical analysis model for the dynamic head impact test using the equivalent model: (**a**) overall modeling; (**b**) equivalent modeling in the direction of thickness.

**Figure 13:**
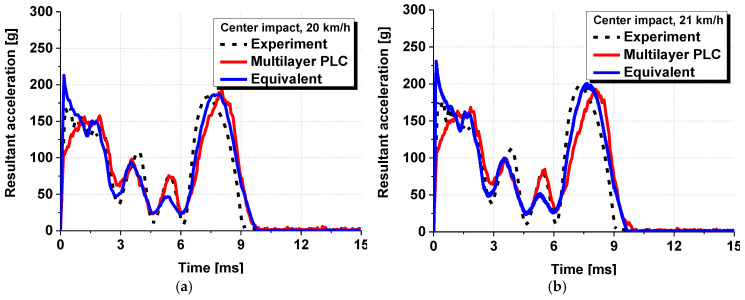

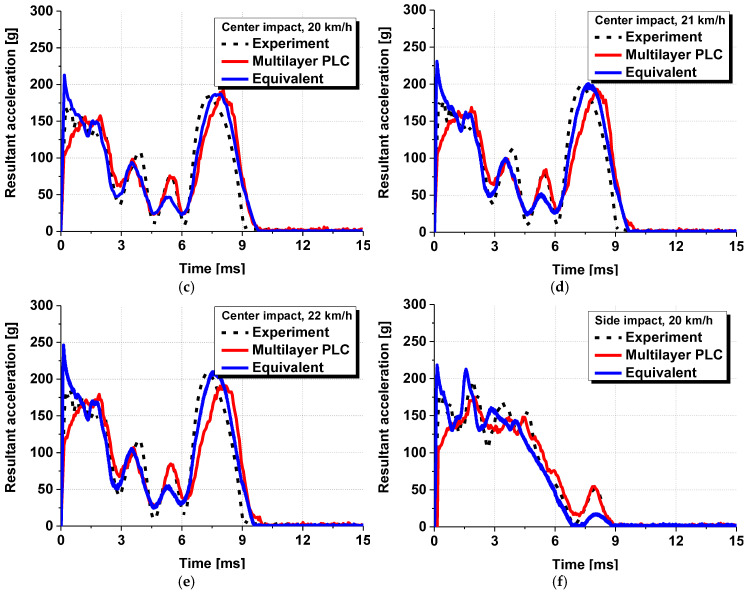
Analytical results of the dynamic head impact test using the equivalent model: (**a**) center impact at a speed of 20 km/h; (**b**) center impact at 21 km/h; (**c**) center impact at 22 km/h; (**d**) side impact at 20 km/h; (**e**) side impact at 22 km/h; (**f**) side impact at 24 km/h.

A numerical analysis identical to the one conducted in Section 3.1 was conducted by applying the equivalent model proposed in this study. Figure 12 shows the numerical analysis model with the equivalent model applied, in which the PVB-laminated glass was modelled as a single layer. All other analysis conditions remained the same, and six cases from the previous analysis were conducted repeatedly. Figure 13 presents the time–acceleration graphs measured from the head dummy to depict the results of the actual dynamic head impact test, PLC analysis model, and equivalent model. The results demonstrated that the acceleration tendency of the equivalent model closely matched the experimental results and those of the existing PLC model. The trends aligned under various experimental conditions. Moreover, in terms of analysis cost, the PLC model required approximately 8 h to complete the analysis, whereas the equivalent model reduced the analysis time to less than 1 h for the same case, thus, demonstrating a dramatic decrease in the analysis cost.

## 4. Conclusions

In this study, an equivalent model of PVB-laminated glass is proposed to simulate collision behavior. The PLC model replicates the multi-layer structure of PVB-laminated glass, including tempered glass and PVB film. However, the proposed equivalent model simplifies this by modeling tempered glass and PVB film as a single shell element layer, incorporating the multi-layer characteristics via integration points. This simplification maintains analytical equivalency with the PLC model. Consequently, the equivalent model produces results comparable to the PLC model while significantly reducing analysis time from 8 h to 1 h under the same hardware and software conditions. Although the proposed equivalent model cannot analyze the fracture pattern of PVB-laminated glass, it demonstrates high computational efficiency at impact speeds that do not cause glass fracture. Thus, it is suitable as an analytical model for low-speed vehicles such as TRAMs. Particularly for TRAMs, which are currently in the introduction and operational stages, the demand for computational analysis for front glass design is expected to be significant. By applying the proposed method to the design of TRAMs’ front windshields, which are increasingly being adopted, the behaviors of pedestrian impacts can be reliably and quickly analyzed to significantly reduce the time required for front-end design in the initial design phase of a TRAM.

## Figures and Tables

**Table 1:** Material constants of the Mooney–Rivlin fitting equation with respect to the strain rate.

	1/s	10/s	100/s	200/s	400/s
$A_{1}$	−0.295	−1.027	−4.935	−4.962	−10.984
$A_{2}$	1.157	3.451	10.793	11.359	20.835
$A_{3}$	0.073	0.216	0.614	0.562	1.212

## Data Availability

Data are contained within the article.
